# Suprascapular Notch Variations in Dry Human Scapulae: Implications for Suprascapular Nerve Entrapment

**DOI:** 10.7759/cureus.101981

**Published:** 2026-01-21

**Authors:** Parul Upadhayay, Ranjeeta Hansdak, Nikhil Aggarwal, Sneh Agarwal

**Affiliations:** 1 Anatomy, Army College of Medical Sciences, New Delhi, IND; 2 Anatomy, Lady Hardinge Medical College, New Delhi, IND

**Keywords:** anatomical variation, scapula, shoulder pain, suprascapular nerve, suprascapular notch

## Abstract

Introduction

The suprascapular notch (SSN) is an important anatomical landmark along the upper border of the scapula, which allows the suprascapular nerve to pass through. Variations in the morphology of the SSN, particularly those associated with ossification of the superior transverse scapular ligament (STSL), are known to predispose individuals to suprascapular nerve entrapment. Knowledge of these variations is therefore essential for accurate diagnosis and safe surgical interventions around the shoulder region.

Aim

To study the morphological variations of the suprascapular notch in dry adult human scapulae.

Materials and methods

The present descriptive study was conducted on 70 dry adult human scapulae of unknown age and gender acquired from the osteology collection of the Anatomy Department, Lady Hardinge Medical College, New Delhi. Scapulae with damaged or deformed superior borders were excluded. The SSN of each specimen was carefully examined and classified into Types I-VI based on the modified Rengachary classification. The frequency and percentage distribution of each notch type were recorded. Statistical analysis was performed using SPSS by applying the Z-test for a single proportion for each type of suprascapular notch, statistical p value of less than 0.05 regarded as significant.

Results

Among the 70 scapulae studied, Type III suprascapular notch was the most common, observed in 40 (57.10%) specimens. This was followed by Type VI in 11 (15.71%), Type I in 10 (14.28%), Type II in 5 (7.14%), and Type IV in 4 (5.71%) speciments. Type V suprascapular notch was not observed (0, 0%) in the present study. The distribution of suprascapular notch types was found to be statistically significant (p<0.05).

Conclusion

The current study reveals that Type III suprascapular notch is the predominant morphological pattern. Variations involving narrowing of the notch or transverse scapular ligament ossification may likely lead to suprascapular nerve entrapment. Awareness of these anatomical variations is clinically important for orthopaedic surgeons, radiologists, and clinicians involved in the evaluation and management of shoulder pathologies.

## Introduction

The shoulder blade or scapula is a flat bone that connects the upper limb to the axial skeleton and plays a crucial role in providing stability and facilitating a wide range of movements at the shoulder joint. Among the key anatomical landmarks of the scapula, the suprascapular notch (SSN) is located on the superior border, positioned just medial to the base of coracoid process. The fact that SSN serves as a conduit for the suprascapular nerve, which supplies the muscles of the rotator cuff (supraspinatus and infraspinatus), the SSN becomes clinically significant [1,2].

The suprascapular nerve carries fibres from the C5 and C6 spinal nerve roots and arises from the upper trunk of the brachial plexus. It then passes beneath the transverse scapular ligament through the suprascapular foramen, while the suprascapular vessels typically course superior to it. Any alteration in the morphology of the scapular notch or ossified superior transverse scapular ligament (STSL), can significantly reduce the available space for the nerve and predispose it to compression [1,3,4]. This anatomical relationship makes the suprascapular notch one of the most common sites of suprascapular nerve entrapment [5].

Andre Thomas was among the earliest authors to describe suprascapular nerve entrapment in 1936 and subsequent studies have further substantiated this condition through histological and immunohistochemical evidence demonstrating nerve fibre degeneration in cases where the superior transverse scapular ligament is ossified [2,6]. Ossified STSL increases the risk of nerve compression by converting the scapular notch into a foramen or hiatus [7,8].

Clinically, suprascapular nerve entrapment typically presents as a vague yet persistent pain over the posterior and lateral aspects of the shoulder [9]. This is usually associated with impairment of lateral rotation or abduction of shoulder joint owing to involvement of the supraspinatus and infraspinatus muscles. Long-standing compression can lead to muscle atrophy and functional impairment, often mimicking rotator cuff pathology. Such symptoms are frequently encountered in individuals involved in repetitive overhead activities, including athletes such as volleyball players, as highlighted in previous anatomical and clinical studies [2,3,10,11].

From a developmental standpoint, the scapula develops from multiple ossification centres derived from paraxial mesoderm and neural crest cells. Variations in suprascapular notch morphology may arise due to alterations in the timing, fusion, or signalling mechanisms during the ossification process [12]. The superior transverse scapular ligament develops from local mesenchymal condensation, and its partial or complete ossification has been attributed to developmental programming as well as mechanical stressors such as repetitive microtrauma and sustained overhead activity [3,6]. Genetic predisposition and local vascular patterns have also been proposed as contributing factors to the observed population-specific variability in notch morphology [1].

Several classifications of the suprascapular notch have been proposed over time. Early descriptions by Hrdlička and Olivier broadly categorised the notch based on its depth and general appearance [13,14]. Hrdlička classified the suprascapular notch into shallow, medium, and deep types, while Olivier described additional morphological variants [1]. Subsequently, Rengachary and colleagues proposed a more detailed classification system that categorised the suprascapular notch into six types, ranging from lack of a notch to fully ossified transverse scapular ligament [15]. Although Ticker et al. [16] and Bayramoğlu et al. [17] altered the above classification, it still remains one of the most widely used systems due to its anatomical clarity and clinical relevance [1,6].

The importance of accurately identifying suprascapular notch variations extends beyond anatomical interest. For orthopaedic surgeons, neurosurgeons, radiologists, and anaesthesiologists, a clear understanding of these variations is essential during shoulder arthroscopy, suprascapular nerve decompression, image interpretation, and nerve block procedures. Failure to recognise high-risk notch configurations may result in iatrogenic nerve injury or misinterpretation of clinical symptoms [2,3].

Although several studies have documented suprascapular notch morphology in different populations, considerable variation exists in the reported prevalence of individual notch types. Such differences emphasise the need for region-specific anatomical data. The present study was therefore undertaken to document the structural and gross variants in the scapular notch of human scapulae, particularly with respect to suprascapular nerve entrapment and understand their other potential clinical implications.

## Materials and methods

The present cross-sectional observational osteological analysis was performed and accomplished in the anatomy department of Lady Hardinge Medical College, New Delhi, during the period from July 2025 to October 2025. Seventy dry, fully developed human scapulae of anonymous and unspecified age and gender were included in the study. The exact demographic details of the specimens were not available as all specimens were obtained from the departmental osteology collection and were routinely used for teaching and academic purposes. As the study involved dry human bones from an established osteology collection, no living subjects were involved and no personal identifiers were available. All specimens were handled respectfully and in accordance with institutional ethical guidelines governing the use of human skeletal material for academic and research purposes.

Scapulae showing any gross morphological deformity, fracture, damage, or irregularity involving the superior border were excluded from the study to prevent misinterpretation of suprascapular notch morphology.

Each scapula was carefully examined for the presence and morphological characteristics of the suprascapular notch located on the superior border of the scapula, medial to the root of the coracoid process. The specimens were cleaned and observed under adequate illumination, and careful visual inspection was carried out to identify the contour of the notch and the condition of the ligamentum transversum scapulae superius (Latin for STSL). Special attention was given to the margins of the notch to detect narrowing, widening, or ossification of the ligament that could influence the morphology of the scapular notch.

Classification of scapular notch in each scapula was done on the basis of modified Rengachary system [15]. According to this classification, Type I is characterised by the absence of a discrete suprascapular notch, presenting as a wide depression on the upper border of scapula. Blunt V-shaped notch in the middle third of the upper border is often described as Type II. Type III is a symmetrical U-shaped notch having parallel lateral borders. Type IV is a narrow and deep V-shaped notch with reduced transverse width. Type V represents a suprascapular notch associated with fractional and limited ossified transverse scapular ligament. Type VI is characterised by fully ossified transverse scapular ligament, resulting in the conversion of the suprascapular notch into a bony foramen. Each scapula was examined meticulously and categorised into one of these six types based on its gross anatomical appearance.

The observed suprascapular notch type for each specimen was recorded systematically. The frequency and percentage distribution of the various suprascapular notch types were calculated and tabulated. To determine whether the observed distribution differed significantly from an expected equal distribution among the six categories, statistical analysis was performed using the Z-test for proportions. The Z-value was calculated using the standard formula incorporating the observed proportion, expected proportion, and total number of scapulae examined. Statistical significance was defined as a p-value of less than 0.05.

Statistical analysis was performed using IBM SPSS Statistics for Windows, Version 26.0 (IBM Corp., Armonk, NY, USA) and all calculations were cross-verified using Microsoft Excel (Microsoft, Redmond, WA) to minimise computational errors and ensure accuracy of results. The findings were expressed in terms of absolute numbers, percentages, Z-values, and corresponding p-values.

## Results

A total of 70 dry adult human scapulae were examined to assess the morphological variations of the suprascapular notch. All specimens were suitable for evaluation, as scapulae with damaged or deformed superior borders had been excluded during the selection process. The suprascapular notch was identified in all scapulae and classified into six types based on the modified Rengachary system [15].

The analysis revealed a clear predominance of one morphological pattern over the others. Type III suprascapular notch was the most frequently observed variant, being present in 40 scapulae, accounting for 57.10% out of the entire sample size. This suggests that a symmetrical U-shaped notch with parallel borders was present in almost half of the scapulae that were analysed. The next most common type was Type VI, observed in 11 scapulae (15.71%), representing totally ossified transverse scapular ligament, thereby converting scapular notch into a bony foramen. Type I suprascapular notch, characterised by the absence of a discrete notch and presence of a wide depression along the superior border, was noted in 10 scapulae (14.28%). Type II, presenting as a blunt V-shaped notch, was observed in five scapulae (7.14%), while Type IV, a narrow and deep V-shaped notch, was identified in four scapulae (5.71%). Notably, Type V suprascapular notch, associated with partial ossification of the superior transverse scapular ligament, was not observed in any of the examined specimens. The overall frequency and percentage distribution of the different suprascapular notch types are summarised in Table 1, which demonstrates a markedly uneven distribution among the six morphological categories. This uneven distribution highlights the dominance of Type III morphology in the present sample, along with a considerable proportion of Type VI notches, which are clinically relevant due to their association with suprascapular nerve compression.

**Table 1:** Frequency and percentage distribution of suprascapular notch types in dry human scapulae (n = 70) SSN: Suprascapular notch.

S. No.	SSN Type	Number (n)	Percentage (%)
1	Type I	10	14.28
2	Type II	5	7.14
3	Type III	40	57.10
4	Type IV	4	5.71
5	Type V	0	0
6	Type VI	11	15.71
	Total	70	100.00

**Figure 1:**
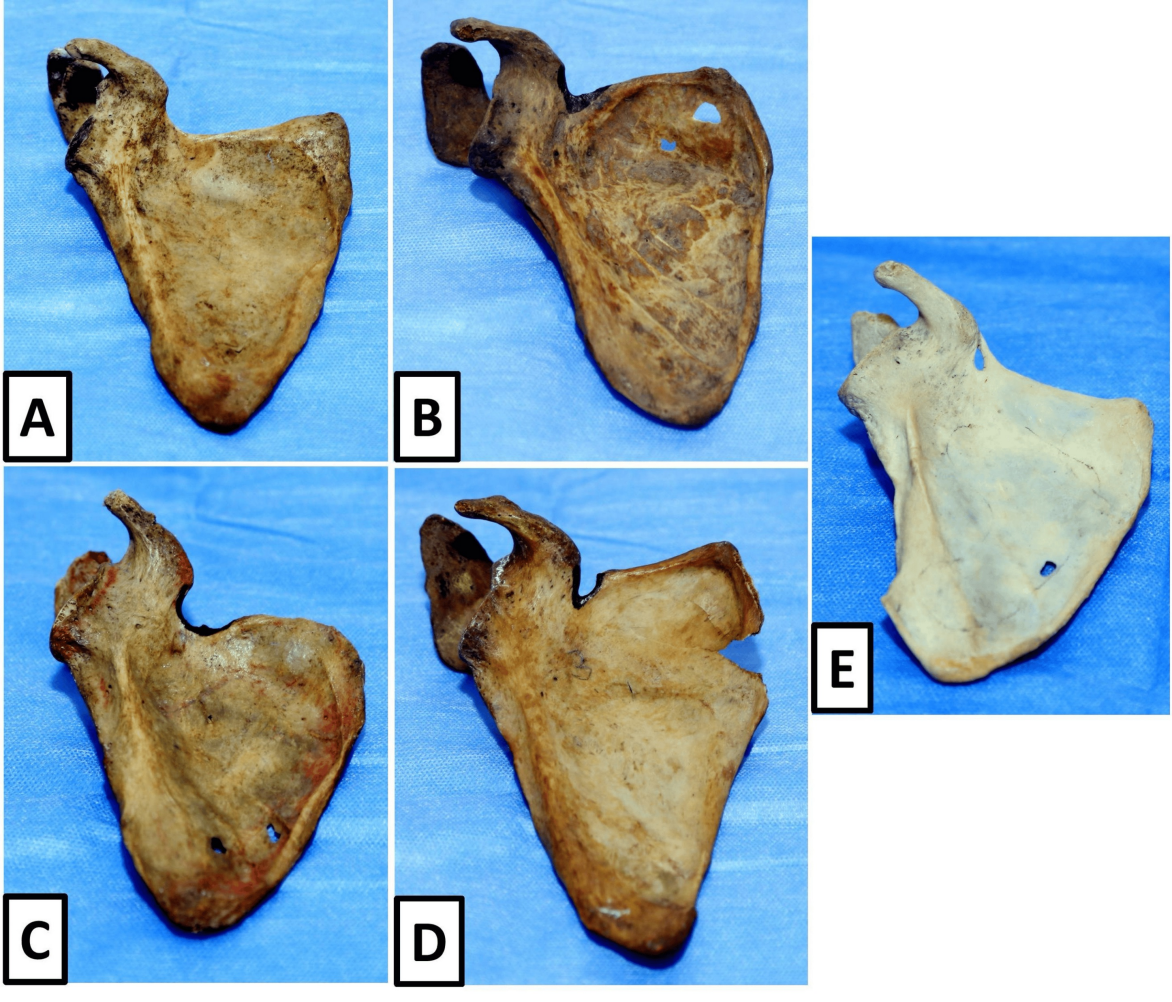
Morphological variations of the suprascapular notch in dry human scapulae. (A) Type I: absence of a distinct suprascapular notch presenting as a wide depression; (B) Type II: unsharpened V-shaped scapular notch; (C) Type III: symmetrical U-shaped suprascapular notch having parallel borders; (D) Type IV: narrow and deep V-shaped suprascapular notch; (E) Type VI: completely ossified transverse scapular ligament forming a suprascapular foramen.

The morphological variations of the suprascapular notch observed in the present study are visually represented in Figure 1. These images provide a visual correlation with the classification criteria used and support the morphological categorisation recorded during observation.

To assess the statistical significance of the observed distribution of suprascapular notch types, a Z-test for proportions was applied, assuming an expected uniform distribution across all six types. The results of this analysis are presented in Table 2. The findings revealed that Type III suprascapular notch showed a highly significant deviation from the expected proportion, with a Z-value of 7.93 and p-value less than 0.0001, indicating a strong predominance of this type in the studied sample. Type II and Type IV notches also demonstrated statistically significant deviations from the expected distribution, with p-values of 0.0307 and 0.0134, respectively, reflecting their relatively lower occurrence. Type V, which was absent in the sample, showed a highly significant negative deviation from the expected proportion. In contrast, Type I and Type VI suprascapular notches did not show statistically significant deviation from the expected distribution, indicating that their observed frequencies were closer to the expected values.

**Table 2:** Z-test for proportions showing statistical significance of distribution of suprascapular notch types

SSN Type	Observed (x)	p = x/70	Z-value	p-value	Significance (p<0.05)
Type I	10	0.1429	-0.46	0.6478	Not significant
Type II	5	0.0714	-2.16	0.0307	Significant
Type III	40	0.5714	7.93	<0.0001	Highly significant
Type IV	4	0.0571	-2.47	0.0134	Significant
Type V	0	0.0000	-3.51	0.0004	Highly significant
Type VI	11	0.1571	-0.24	0.8108	Not significant

Overall, the results of the present study demonstrate a significant variation in the distribution of suprascapular notch morphology, with a clear predominance of Type III and a notable presence of Type VI notches. These findings underscore the anatomical diversity of the suprascapular notch and provide a quantitative basis for further discussion regarding their potential clinical implications, particularly in relation to suprascapular nerve entrapment.

## Discussion

The suprascapular notch is a key anatomical feature of the scapula, as it forms a confined osteoligamentous tunnel for the suprascapular nerve below the transverse scapular ligament. Differences in the morphology of the suprascapular notch and the ossification status of the ligament have long been recognised as important contributors to suprascapular nerve entrapment.The present study aimed to document these variations in dry human scapulae and to interpret their anatomical and clinical relevance in light of the existing literature.

In the present study, Type III suprascapular notch was the most frequently observed morphology, accounting for 40 (57.10%) of the examined scapulae. This finding is in accordance with the observations made via Rengachary et al. and Bayramoğlu et al., who also reported Type III or U-shaped notches as the predominant pattern in their respective populations [15,17]. The relatively wide configuration and parallel margins of a Type III notch are believed to offer a larger passage for the suprascapular nerve, thereby reducing the likelihood of compression. Similar predominance of U-shaped notches has also been documented in more recent studies, including those by Premakumari et al. [6] and Sankat Mochan et al. [18], reinforcing the view that this morphology represents the most common and anatomically favourable variant across populations [6,18].

The second most common finding in the present study was Type VI suprascapular notch, observed in 11 (15.71%) scapulae. Type VI is characterised by entirely and wholly ossified transverse scapular ligament, converting suprascapular notch into a rigid bony foramen. This configuration is of particular clinical importance, as ossification of the ligament eliminates the ligament’s inherent elasticity and reduces the adaptability of the tunnel during shoulder movements. Andre Thomas was among the earliest to describe suprascapular nerve entrapment, and subsequent histological and immunohistochemical studies have demonstrated nerve fibre degeneration in cases associated with ossified superior transverse scapular ligaments [19,20]. Later authors, including Bagoji et al. and Sathiya et al., have also emphasised that complete ossification is one of the strongest anatomical risk factors for suprascapular neuropathy [2,3]. The relatively higher proportion of Type VI notches observed in the present study highlights the need for clinicians to remain vigilant when evaluating patients with unexplained posterior shoulder pain.

Type I suprascapular notch, characterised by the absence of a discrete notch and the presence of a broad depression along the superior border of the scapula, was identified in 10 (14.28%) specimens. This finding is comparable with observations reported by Sankat Mochan et al. and earlier descriptions by Hrdlička, who classified shallow or absent notches as a distinct morphological entity [13,18]. Although Type I notches are generally considered less likely to cause nerve compression due to the absence of a constricting tunnel, their presence remains surgically relevant. Altered bony contours may modify the expected course of the suprascapular nerve and pose challenges during surgical exposure or nerve block procedures.

Type II (5, 7.14%) and Type IV (4, 5.71%) suprascapular notches were less frequently encountered in the present study, together accounting for approximately nine specimens. These notches are typically V-shaped, with Type IV being narrower and deeper than Type II. Several authors, including Talokar et al. and Bagoji et al., have highlighted that narrow V-shaped notches may predispose the suprascapular nerve to compression due to reduced transverse diameter [1,2]. Sathiya et al. further noted that even in the absence of ligament ossification, a narrow notch with reduced depth can increase the risk of neuropathy, particularly during repetitive overhead movements [3]. Although these notch types were relatively uncommon in the present sample, their recognised association with nerve entrapment underscores their clinical significance.

Interestingly in the present research, Type V suprascapular notch, associated with partial ossification of the superior transverse scapular ligament, could not be perceived . This contrasts with findings reported by and Sathiya et al. and Premakumari et al., who documented partial ossification in a proportion of their samples [3,6]. The absence of Type V notches in the current series highlights inter-population variability and suggests that patterns of ligament ossification may be influenced by genetic, developmental, and biomechanical factors. Such variability further supports the need for regional anatomical studies rather than reliance on pooled data alone.

The embryological basis of suprascapular notch variation has been discussed by several authors and was also addressed in the present study. The scapula develops from multiple ossification centres derived from paraxial mesoderm and neural crest cells, and subtle alterations in the timing and pattern of ossification may influence the final morphology of the suprascapular notch [12]. The superior transverse scapular ligament arises from local mesenchymal condensation, and its partial or complete ossification has been attributed to developmental programming as well as external stressors such as repetitive microtrauma and sustained overhead activity. Local vascular factors and genetic predisposition have also been proposed as contributing elements, as discussed by earlier investigators and supported by recent morphometric studies [1,3,4,21].

Several classification systems for the suprascapular notch have been proposed over the years. Early classifications by Hrdlička and Olivier broadly categorised the notch based on depth and general shape, laying the foundation for later, more detailed systems [13,14]. The classification proposed by Rengachary et al., which was utilised in the present study, remains one of the most widely accepted due to its anatomical clarity and clinical applicability [15]. However, Yiannakopoulos et al. reported significant inter-observer and intra-observer variability among commonly used classification systems, highlighting the need for careful anatomical assessment and clear descriptive criteria when categorising suprascapular notch morphology [22]. Despite these limitations, the Rengachary system continues to provide a practical framework for correlating anatomical variations with clinical risk. Table 3 summarises the frequency distribution of suprascapular notch types as reported in previous studies from different populations, arranged in chronological order, and compares them with the findings of the present study.

**Table 3:** Comparison of different types of suprascapular notch reported by various authors across different populations , arranged in chronological order.

S. No.	Author (Year)	Region	Sample size (n)	Type I (%)	Type II (%)	Type III (%)	Type IV (%)	Type V (%)	Type VI (%)
1.	Rengachary et al. (1979) [15]	Original series	211	8.00	31.00	48.00	3.00	6.00	4.00
2.	Bayramoğlu et al. (2003) [17]	Turkish population	93	6.4	19.4	50.5	7.5	9.7	6.5
3.	Natsis et al. (2006) [23]	Greece	423	3.55	8.75	61.94	7.8	10.16	7.8
4.	Das et al. (2007) [24]	North India	100	5.00	13.00	58.00	7.00	11.00	6.00
5.	Kannan et al. (2014) [21]	Puducherry	400	20.00	10.00	52.00	4.00	4.00	10.00
6.	Bagoji et al. (2020) [2]	Karnataka	138	5.07	21.01	48.55	-	-	4.34
7.	Talokar et al. (2023) [1]	Central India	100	26.00	28.00	34.00	8.00	4.00	0.00
8.	Sankat Mochan et al. (2024) [18]	New Delhi	120	15.0	5.83	54.16	-	-	4.16
9.	Sathiya et al. (2024) [3]	South India	50	24.00	16.00	24.00	20.00	12.00	4.00
10.	Premakumari et al. (2025) [6]	Karnataka	120	6.67	7.50	71.50	0.80	8.30	5.80
11.	Present study (2025)	Lady Hardinge Medical College, New Delhi	70	14.28	7.14	57.10	5.71	0.00	15.71

From a clinical perspective, the findings of the present study hold relevance for orthopaedic surgeons, neurosurgeons, radiologists, and anaesthesiologists. Knowledge of the prevalence of various suprascapular notch types, particularly those associated with narrowing or ligament ossification, is essential during shoulder arthroscopy, suprascapular nerve decompression, and image-guided nerve block procedures [10,11,16]. Radiologists must also be aware of these variations to avoid misinterpretation of imaging findings and to correlate anatomical features with clinical symptoms more accurately.

In summary, the present study demonstrates considerable variation in suprascapular notch morphology, with a predominance of Type III notches and a notable proportion of Type VI notches. While the predominance of Type III suggests a relatively favourable anatomical pattern in the studied sample, the presence of ossified ligaments in a significant number of scapulae underscores the importance of anatomical awareness in clinical practice. These findings add to the existing literature by providing region-specific data and reinforce the need for meticulous evaluation of suprascapular notch morphology in both anatomical research and clinical settings.

Limitations of the study

The present study was conducted on a limited number of human scapulae whose age and gender were not known, which restricted assessment of age- and sex-related variations in suprascapular notch morphology. As the specimens were obtained from a single institutional osteology collection, the findings may not fully represent broader population diversity. Being a purely osteological study, radiological and clinical correlations with suprascapular nerve entrapment could not be established, and evaluation of soft tissue components such as the superior transverse scapular ligament in non-ossified cases was not possible. Additionally, despite the use of modified Rengachary system, minor observer-dependent variability in morphological categorisation cannot be completely excluded.

## Conclusions

The present study demonstrates that the suprascapular notch exhibits considerable morphological variation, with Type III being the most prevalent configuration in the examined scapulae. However, a significant proportion of scapulae showed a fully ossified transverse scapular ligament, leading to Type VI suprascapular notch, which is recognised as a possible cause for suprascapular neuropathy. These findings underscore the importance of detailed anatomical knowledge of suprascapular notch variations for clinicians involved in the evaluation and management of shoulder disorders. Awareness of such variations is particularly relevant for orthopaedic surgeons, radiologists, and anaesthesiologists during diagnostic assessment, surgical intervention, and nerve block procedures. The study provides valuable region-specific anatomical data and reinforces the need for careful consideration of suprascapular notch morphology in both anatomical research and clinical practice.
